# Magnetoreception in birds: I. Immunohistochemical studies concerning the cryptochrome cycle

**DOI:** 10.1242/jeb.110965

**Published:** 2014-12-01

**Authors:** Christine Nießner, Susanne Denzau, Leo Peichl, Wolfgang Wiltschko, Roswitha Wiltschko

**Affiliations:** 1 Goethe-Universität Frankfurt, FB Biowissenschaften, Max-von-Laue-Straße 13, D-60438 Frankfurt am Main, Germany; 2 Max-Planck-Institute for Brain Research, Max-von-Laue-Straße 4, D-60438 Frankfurt am Main, Germany

**Keywords:** Magnetic compass, Cryptochrome 1a, Flavin cycle, Photoreduction, Activated Cry1a, Radical pair mechanisms

## Abstract

Cryptochrome 1a, located in the UV/violet-sensitive cones in the avian retina, is discussed as receptor molecule for the magnetic compass of birds. Our previous immunohistochemical studies of chicken retinae with an antiserum that labelled only activated cryptochrome 1a had shown activation of cryptochrome 1a under 373 nm UV, 424 nm blue, 502 nm turquoise and 565 nm green light. Green light, however, does not allow the first step of photoreduction of oxidized cryptochromes to the semiquinone. As the chickens had been kept under ‘white’ light before, we suggested that there was a supply of the semiquinone present at the beginning of the exposure to green light, which could be further reduced and then re-oxidized. To test this hypothesis, we exposed chickens to various wavelengths (1) for 30 min after being kept in daylight, (2) for 30 min after a 30 min pre-exposure to total darkness, and (3) for 1 h after being kept in daylight. In the first case, we found activated cryptochrome 1a under UV, blue, turquoise and green light; in the second two cases we found activated cryptochrome 1a only under UV to turquoise light, where the complete redox cycle of cryptochrome can run, but not under green light. This observation is in agreement with the hypothesis that activated cryptochrome 1a is found as long as there is some of the semiquinone left, but not when the supply is depleted. It supports the idea that the crucial radical pair for magnetoreception is generated during re-oxidation.

## INTRODUCTION

The Radical Pair Model (Ritz et al., 2000) proposes that the sensing of magnetic direction in birds is based on radical pair processes, with cryptochrome as receptor molecule. Applying radio frequency fields in the MHz range was suggested as a diagnostic tool to identify radical pair processes (Ritz, 2001; Henbest et al., 2004), and such fields indeed disrupted magnetic orientation of European robins, *Erithacus rubecula* (Turdidae), domestic chickens, *Gallus gallus* (Phasianidae), and zebra finches, *Taeniopygia guttata* (Estrildidae) (Ritz et al., 2004; Ritz et al., 2009; Thalau et al., 2005; Wiltschko et al., 2007; Keary et al., 2009). In an immunohistochemical study, a type of cryptochrome, Cry1a, was identified in the retina of robins and chickens, where it is located at the discs in the outer segment of the UV/violet cones (Nießner et al., 2011). These findings support the Radical Pair Model and the role of Cry1a as receptor molecule.

In our immunohistochemical study (Nießner et al., 2011), we had used an antiserum against Cry1a recognizing an antigen sequence at the C-terminal region of the protein. In a subsequent study (Nießner et al., 2013), it became evident that this antiserum labelled only the light-activated form of Cry1a: in the non-activated state, the antigen site of the antiserum seems to be hidden; light activation appears to lead to a conformational change that exposes the C-terminus and thus allows our antiserum to bind. This finding gave us the opportunity to study the activation characteristics of Cry1a *in vivo* under various wavelengths of narrow-band light, the same light we had also used in previous behavioural tests. We found activated Cry1a under all wavelengths where birds had been shown to be able to use their magnetic compass (see Wiltschko et al., 2010): 373 nm UV, 424 nm blue, 502 nm turquoise and 565 nm green light (Nießner et al., 2013).

**Fig. 1.**
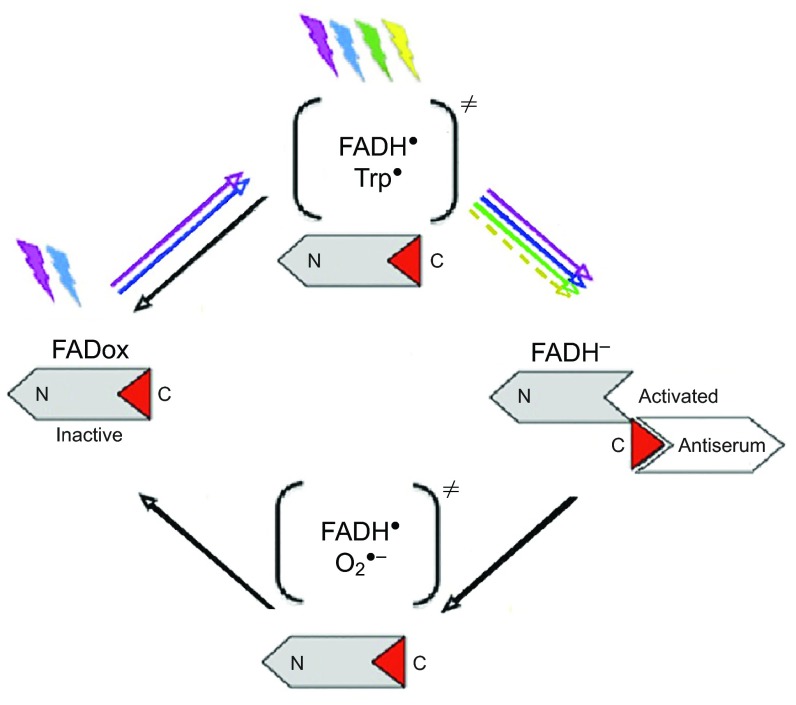
**Flavin cycle of cryptochrome.** N, N-terminus of the protein; C, C-terminus of the protein; the antiserum-binding epitope is in red, indicating that the antiserum probably binds the fully reduced FADH^−^. Radical pairs are shown in parentheses; coloured arrows indicate light-driven reactions, black arrows indicate light-independent reactions (from Nießner et al., 2013).

Cryptochrome is a blue light receptor, with flavin as the chromophore (for review, see Chaves et al., 2011). Flavin undergoes a redox cycle (see Fig. 1): the fully oxidized form, FADox, is photoreduced by UV and blue light up to about 500 nm to the semiquinone FADH^•^ that forms a first radical pair FADH^•^/Trp^•^ with tryptophan. It can be re-oxidized directly in a light-independent reaction or, if light is present, it can absorb UV, blue and green light up to about 570 nm to be further reduced to the fully reduced form, FADH^−^. This fully reduced form of flavin is re-oxidized in a light-independent reaction, generating a second radical pair, possibly FADH^•^/O_2_^•−^ (see Müller and Ahmad, 2011). The observation that labelled Cry1a occurred under green light, but not under red light, seemed to suggest that the fully reduced FADH^−^ is the activated form labelled by our antiserum (Nießner et al., 2013).

In view of this, the activation of Cry1a and the orientation of birds under 565 nm green light are of particular interest. Under UV, blue and turquoise light, the full cycle can run; green light, in contrast, cannot initiate the first step of photoreduction from the fully oxidized form to the semiquinone FADH^•^. Nevertheless, we had found activated Cry1a after 30 min of illumination with green light (Nießner et al., 2013). In this case, the birds had previously been kept in daylight where the entire cycle can run, and so we argued that when the exposure to green light began, there had been a certain supply of FADH^•^ present, which could then be photoreduced to the fully reduced form. If this were true, we would find activated Cry1a until this supply was used up, which obviously took more than the 30 min exposure.

To test this assumption, we carried out the following experiments, using the identical lights that we had used in the previous immunohistological and behavioural studies: (1) we exposed

chickens, *G. gallus* (Linnaeus 1758), that had been kept under daylight to UV, blue, turquoise and green light for 30 min; (2) we exposed birds that had been kept for 30 min in total darkness to light of these wavelengths for 30 min; and (3) we exposed birds that had been kept in daylight to light of these wavelengths for the longer time of 60 min (see Table 1).

Here, we report the results of the immunohistochemical study. The results of corresponding behavioural experiments are reported in the accompanying paper (Wiltschko et al., 2014).

## RESULTS

**Fig. 2.**
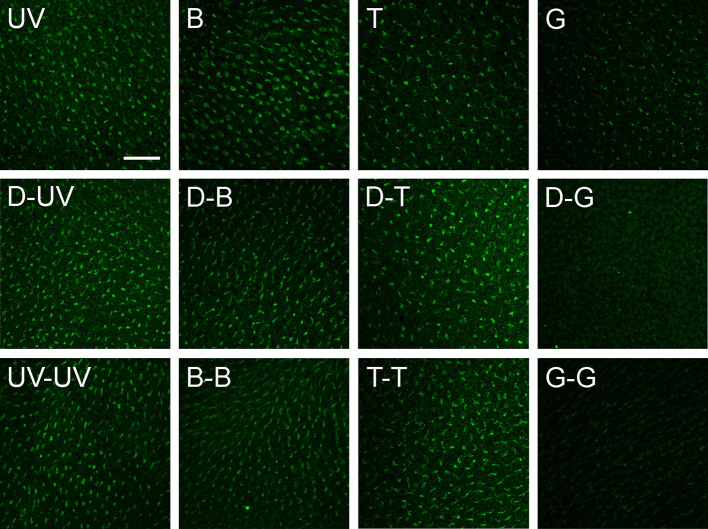
**Views of the photoreceptor layer (cone outer segments) in flattened chicken retinae immunolabelled with Cry1a antiserum.** Our Cry1a antiserum detects the activated form of Cry1a present in the outer segments of the violet-sensitive cone type. The micrographs show the amount of activated Cry1a after exposure of the chickens to narrow bandwidth light of various wavelengths: UV, 373 nm ultraviolet; B, 424 nm blue; T, 502 nm turquoise; and G, 565 nm green (see Table 1). Top row: 30 min exposure to the respective light after being kept in daylight; middle row: 30 min exposure after being kept for 30 min in total darkness (D); bottom row: 60 min exposure to the respective light after being kept in daylight. Scale bar, 50 μm for all panels.

The results of the different treatments with various pre-treatments and wavelengths are given in Fig. 2. The control labelling with antiserum against the SWS1 opsin was clearly visible in all cases (see supplementary material Fig. S1): it was not affected by the different light regimes. As in our previous studies (Nießner et al., 2011; Nießner et al., 2013), the Cry1a label was restricted to the violet-sensitive cones that were identified by SWS1 co-labelling.

In the chicks that had been kept in daylight and then exposed for 30 min to the test lights, we found activated Cry1a at all wavelengths, although the amount of Cry1a after exposure to green light was markedly less than that after exposure to UV, blue and turquoise light (Fig. 2, top row). The retinae shown here are different from those presented in our previous paper (Nießner et al., 2013), yet the results are identical.

Fig. 2, middle row, gives the results for the chickens that were first kept for 30 min in total darkness, and subsequently exposed for 30 min to the test lights. Under UV, blue and turquoise light, there was a considerable amount of activated, labelled Cry1a, but no labelled Cry1a was found under green light.

In the last part of the study, the chickens, after being kept with access to daylight, were exposed to the test light for 60 min – this is equivalent to a 30 min pre-exposure to the same light as used in a 30 min exposure. The results are given in Fig. 2, bottom row: again, there is activated Cry1a labelled under UV, blue and turquoise light, but not under green light.

In summary, green light cannot activate Cry1a after exposure to total darkness or when the exposure to green light lasts for a long time.

## DISCUSSION

Our findings are in agreement with the assumption (see Nießner et al., 2013) that the activation of Cry1a under green light can occur as long as there is a supply of the semiquinone FADH^•^ available. The first step of photoreduction leads to the formation of the radical pair FADH^•^/Trp^•^, the coherence time of which must be assumed to be in the range of microseconds (see Ritz et al., 2009), but the semiquinone FADH^•^ itself appears to be present for a much longer time.

For the experiment with pre-exposure to total darkness, the scenario appears to be as follows. Our previous study had shown that there was no activated Cry1a after 30 min in total darkness (Nießner et al., 2013). The supply of FADH^•^ present at the beginning of the exposure is diminished by the light-independent direct re-oxidation of the flavin (see Fig. 1), and no new FADH^•^ is produced in the dark. After transfer to the test light, any semiquinone left after 30 min is further reduced to the fully reduced form FADH^−^ and then re-oxidized. Under UV, blue and turquoise light, new FADH^•^ is produced, but not under green light. After 30 min exposure to green light, the initial supply of FADH^•^ appears to be depleted, and without new FADH^−^ formed, the existing FADH^−^ is probably re-oxidized so that there is nothing left to be immunolabelled. In UV, blue and turquoise light, in contrast, the full cycle will run, generate new FADH^•^ and subsequently a sufficient amount of FADH^−^ for distinct labelling.

The findings from the experiment with the extended (60 min) exposure can be interpreted in a similar way. After 30 min exposure, we found activated, labelled Cry1a with all four wavelengths, although there was less labelling with green light (see also Nießner et al., 2013). In the present study, after twice that exposure time, there was a marked amount of immunolabelled Cry1a with 373 nm UV, 424 nm blue and 502 nm turquoise light; that is, with all wavelengths that allow the full redox cycle of the flavin to run. With

**Table 1.**
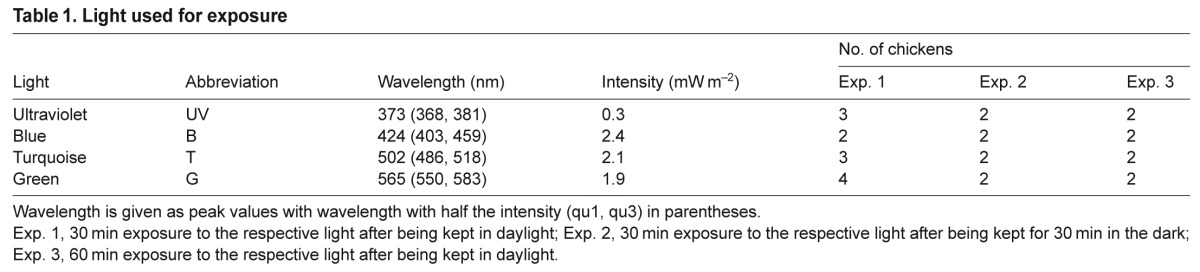
Light used for exposure

green light, which cannot photoreduce oxidized Cry1a and hence cannot produce new semiquinone, the initial supply must be assumed to be converted into the fully reduced form, which, in turn, is re-oxidized in a light-independent reaction. After 60 min, the supply of semiquinone appears to be completely exhausted and the resulting FADH^−^ is already re-oxidized so that no activated Cry1a is left for immunolabelling. With the green light used here and in the behavioural experiments [see accompanying paper (Wiltschko et al., 2014)], a supply of semiquinone produced in daylight seems to last for more than 30 min, but less than 60 min, before it is depleted and the activation of Cry1a stops.

Taken together, the present findings support the interpretation presented in our previous paper (Nießner et al., 2013), namely that the second part of photoreduction is crucial for the activation of Cry1a, with the fully reduced FADH^−^ being the activated form to which our antiserum binds. Suggesting this form as the activated, signalling one is unusual, because in most cryptochromes analysed so far, the conformational change appears to occur during the first step of photoreduction, when the radical FADH^•^/Trp^•^ is generated, which in most cases is considered to be the signalling form (e.g. Berndt et al., 2007; Banerjee et al., 2007; Bouly et al., 2007). Yet, in this case, we would not have found any activated Cry1a under green light. The Cry1a involved in the magnetic compass of birds thus appears to respond somewhat differently from other cryptochromes.

In this context, it has to be considered that the role of Cry1a as receptor molecule for magnetoreception is different from the normal function of cryptochromes, namely signalling the presence or absence and the amount of light. In the avian magnetic compass, cryptochrome has to indicate directions derived from the different singlet/triplet ratio (Ritz et al., 2000). For this task, the fully reduced form FADH^−^ might be more suitable, and it could be rendered magnetically sensitive, because the singlet/triplet ratio of the radical pair formed during re-oxidation, which depends on the alignment of the receptor molecule in the geomagnetic field, could affect the efficiency of re-oxidation [see Müller and Ahmad for a detailed discussion (Müller and Ahmad, 2011)]. If this radical pair generated during re-oxidation is more suitable for indicating direction, and there are reasons to believe that it might be (see Ritz et al., 2009; Lee et al., 2014), evolutionary processes would have shaped the mechanism accordingly and adapted it optimally for the required task.

## MATERIALS AND METHODS

The immunohistochemical study was performed in accordance with the rules and regulations of animal welfare in Germany.

The study involved young domestic chickens about 3 weeks old. At this age, chickens are able to use their magnetic compass, and Cry1a is present in the same amount as in adult chickens (Denzau et al., 2013). The chicks were exposed to narrow-band lights produced by light-emitting diodes (LEDs), with the light level controlled with a radiometer (Optometer P-9710-1, Gigahertz Optik, Puchheim, Germany); wavelengths and intensity are listed in Table 1. First, chicks were treated that had been kept in daylight before; these birds are from the data set already reported (Nießner et al., 2013). Next, we kept chicks for 30 min in total darkness, followed by 30 min of exposure to the respective light. In the last part of the study, chicks that had been maintained in the daylight were exposed to the respective light for 60 min, twice as long as before and in the previous study (Nießner et al., 2013).

Immediately after the end of the exposure, the eyes of the chickens were processed, with the procedure of preparation, fixation and further processing of the retinae exactly following the protocol described in detail in our previous paper (Nießner et al., 2013). The whole isolated retinae were treated with the same guinea pig Cry1a antiserum used in those studies, i.e. with the antiserum that had been demonstrated to label only the light-activated form of Cry1a (see Nießner et al., 2013). As control for proper treatment and marking, we used goat antiserum sc-14363 to immunolabel the violet opsin (SWS1) as in our previous study. Thus, all retinae were incubated in a mixture of the two antisera, and the labelling was visualized with appropriate secondary antibodies coupled to different fluorescent dyes (Cy3 and Alexa 488). The retinae were evaluated with a confocal laser scanning microscope (Zeiss LSM 510 META). Exposure times for the micrographs were equalized to allow direct comparison of the labelling intensities in the different experimental conditions.

Supplementary material Fig. S1 gives the control labelling with the antiserum against violet (SWS1) opsin – this labelling was clearly visible in all cases.

## Supplementary Material

Supplementary Material
