# Supplementary material for: Magnetoreception in birds: I. Immunohistochemical studies concerning the cryptochrome cycle
Source: J Exp Biol. 2014 Dec 1;217(23):4221–4. doi: 10.1242/jeb.110965 (PMC4254396; doi:10.1242/jeb.110965)
Supplement: Supplementary Material [file supp_217.23.4221_JEB110965.pdf]

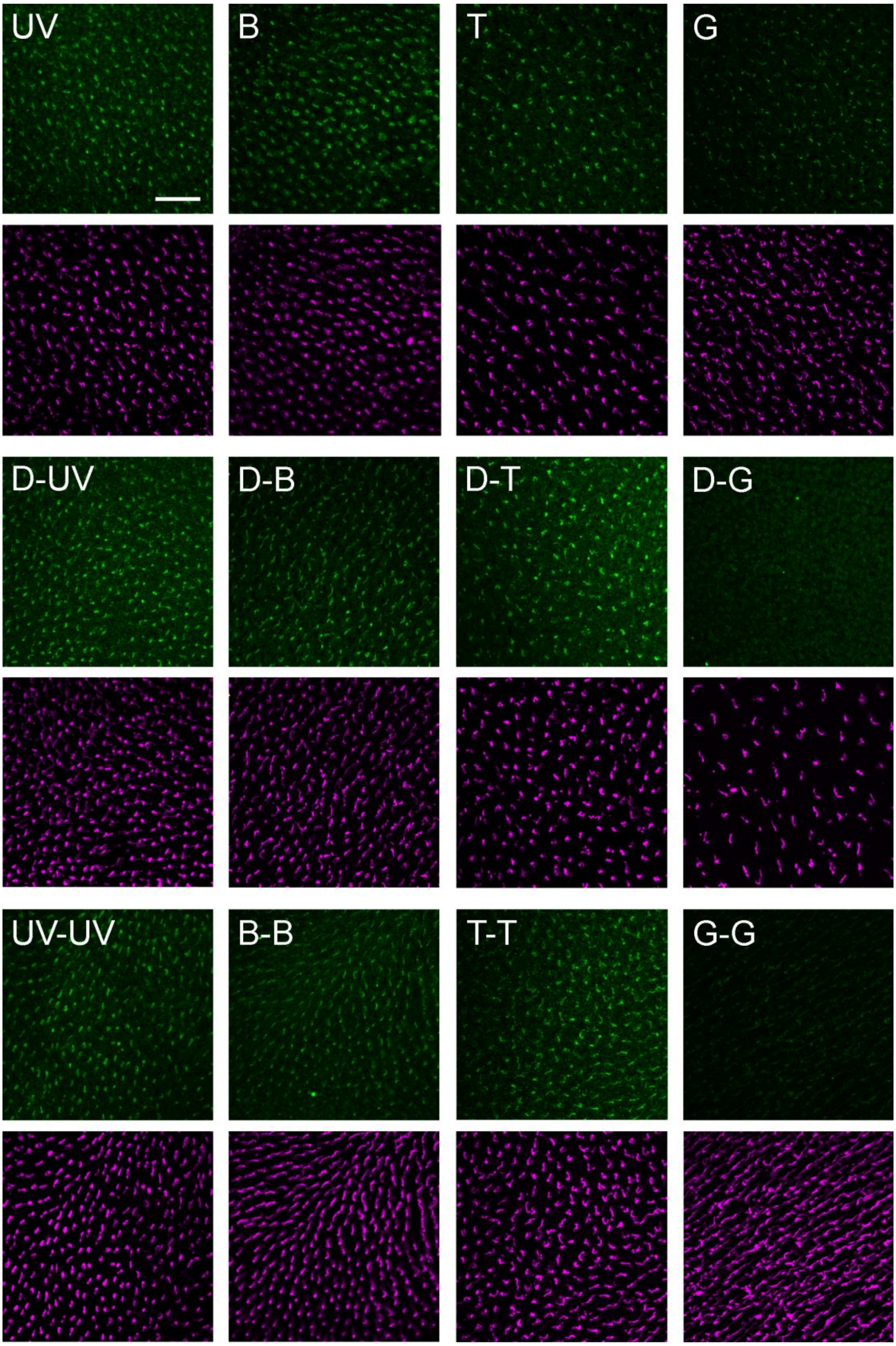

Fig. S1. Control immunolabelling for violet (SWS1) cone opsin. Shown are micrographs of fields in flattened chicken retinae double labelled for Cry1a (green fluorescence) and for the violet opsin (magenta fluorescence). The fields are the same as shown in Fig. 2 of the main text. As described there, labelling was performed after exposure of the animals to narrow bandwidth lights of various wavelengths: UV, 373 nm ultraviolet; B, 424 nm blue; T, 502 nm turquoise; G, 565 nm green. Two top rows: 30 min exposure to the respective light after being kept in daylight; two middle rows: 30 min exposure after being kept for 30 min in total darkness (D-X, with X representing the respective colours); two bottom rows: 60 min exposure to the respective colour lights (X-X) after being kept in daylight. For each condition, the Cry1a image and the opsin image below show the two labels in the same cones in the same field. In contrast to the Cry1a labelling, the opsin labelling is not affected by the different light regimes, showing the integrity of the cones. The field illustrating the D-G condition is from peripheral retina where the violet cone density is low. Scale bar, 50 μm for all panels.
